# Alternative sources of cautery may improve post-operative hematoma rates but increase operative time in thyroid surgery

**DOI:** 10.1038/s41598-021-01953-5

**Published:** 2021-11-19

**Authors:** Corliss A. E. Best, Alexandra E. Quimby, Stephanie Johnson-Obaseki

**Affiliations:** The Department of Otolaryngology-Head and Neck Surgery, The University of Ottawa, 501 Smyth Road, Ottawa, ON K1H 8L6 Canada

**Keywords:** Thyroid diseases, Surgical oncology

## Abstract

A retrospective risk-adjusted analysis was completed using data from the National Surgical Quality Improvement Program (NSQIP) to (1) compare the risks of post-operative hematoma for thyroid surgery using conventional cautery compared alternative energy devices (defined as LigaSure and Harmonic Scalpel), and (2) compare operative times for the same. The primary outcome variable was post-operative hematoma occurrence. The secondary outcome variable was operative time. The exposure variable was use of conventional or alternative sources of cautery. All adult patients who underwent a total thyroidectomy, subtotal thyroidectomy or completion thyroidectomy between 2016 and 2018 were included. Multivariable linear and logistic regression analyses were performed to control for potentially confounding variables. A total of 13,330 cases were analyzed; 4342 used conventional cautery, and 8988 used alternative sources. There was a statistically significant decrease in post-operative hematoma risk using alternative sources of cautery compared to conventional cautery (OR 0.75; 95% CI 0.58–0.98) (p = 0.04). Use of alternative sources of cautery added 4.95 min onto operative time (95% CI 2.45–7.45) which was statistically significant (p < 0.0001). After controlling for confounding variables, there was a statistically significant lower rate of post-operative hematoma in thyroidectomies performed using alternative sources of cautery compared to those performed with traditional hemostatic methods. Alternative sources of cautery increased operative time by 4.95 min.

## Introduction

Meticulous hemostasis is one of the most important goals in thyroid surgery, both for excellent visualization of local vital structures and for prevention of post-operative hematoma^1^. Conventional hemostatic techniques include the use of conventional bipolar and monopolar cautery, ‘clamp-and-tie’ or vascular ligations, and the use of hemostatic clips. These techniques are considered the gold standards for thyroid surgery, although can have potential pitfalls^2^. Traditional clamp-and-tie technique and use of clips can be time consuming or may slip off, while electrocautery runs the risk of heat dispersion and injury to important surrounding structures^3^.

In response to such concerns, alternative sources of cautery have been developed. The aim of these devices is to achieve fast and safe hemostasis with less thermal spread, with the overall goal of reducing operative time and complications^3^. Two alternative sources of cautery widely used in thyroid surgery are the Harmonic Scalpel and the LigaSure Vessel Sealing System.

The Harmonic Scalpel (formerly named UltraCision; Ethicon Endo- Surgery, Cincinnati, OH) uses ultrasound technique that achieves simultaneous cutting and coagulation of blood vessels via mechanical vibration at a frequency of 55.5 kHz^4^. This vibration causes a rupture of protein hydrogen bonds, thereby denaturing the proteins to form a coagulum. The coagulum then seals the vessels and assures hemostasis at ‘low’ temperatures (between 50 and 100 °C)^5^. The manufacturers guarantee that vessels with a diameter less than or equal to 5 mm are sealed using this apparatus^4^. The LigaSure vessel sealing system (Valleylab, Boulder, Colorado) is a method of bipolar hemostasis that coagulates vessels with a diameter less than or equal to 7 mm^2^.

Ample research has been conducted to compare outcomes in thyroid surgery using conventional cautery and such alternative energy devices. The bulk of these studies have focused on operative efficiency (including operative time), intra-operative blood loss, post-operative complications, surgical completeness in oncologic surgery, and cost-effectiveness^6–8^. The device best able to optimize all of the above is still hotly debated.

The vast majority of the studies comparing the effectiveness of hemostasis between devices have used suction drainage outputs as a proxy for potential hematoma collection, or have documented hematoma rates as part of a composite variable of post-operative complications^1,7^. To the authors’ knowledge, there has not been a study comparing these devices with the primary outcome of post-operative hematoma risk. As such, the aim of this study was to use a large, multi-institutional database to compare post-thyroidectomy hematoma as a clinically significant outcome between conventional hemostasis and alternative energy devices (operationally defined as the Harmonic scalpel and LigaSure devices). The secondary outcome of this study was to compare operative time for thyroid surgery using conventional cautery vs alternative sources.

## Materials and methods

### Study design

A retrospective cohort study of patients undergoing total thyroidectomy, subtotal thyroidectomy or completion thyroidectomy was conducted. The primary outcome variable was post-operative hematoma. The secondary outcome variable was operative time. The exposure variable was dichotomized as traditional or alternative sources of cautery. For the purposes of this study, use of “alternative” sources of cautery was defined as use of either of the Harmonic scalpel or the LigaSure device.

Data was collected from the American College of Surgeons National Surgical Quality Improvement Program (ACS-NSQIP) registry from the years 2016–2018, as 2016 marked the first year that thyroid specific data was collected^9^. First developed by the US Department of Veteran Affairs, the ACS-NSQIP database is a comprehensive surgical database and quality improvement program which collects data and provides risk-adjusted outcomes^10^. Details of the ACS-NSQIP database collection methods have been previously described in the literature^11^. This study did not require institution ethical approval as NSQIP data exempt from The Ottawa Hospital REB as it is deidentified. A consent waiver was not applicable as all data is deidentified. The study was conducted in accordance with the NSQIP database regulations.

### Population

Adult patients (> 18 years) were selected for inclusion based on current procedural technology (CPT) codes that were gathered from the ACS-NSQIP database. In order to isolate patients undergoing total thyroidectomy, subtotal thyroidectomy, and completion thyroidectomy, the following CPT codes were included: 60210, Partial thyroid lobectomy, unilateral, with or without isthmusectomy; 60212, Partial thyroid lobectomy, unilateral, with contralateral subtotal lobectomy, including isthmusectomy; 60220, total thyroid lobectomy, unilateral, with or without isthmusectomy; 60225, total thyroid lobectomy, unilateral, with contralateral subtotal lobectomy, including isthmusectomy; and 60240, thyroidectomy, total or complete. We excluded the following CPT codes to control for factors or additional procedures that might significantly affect operative time and post-operative hematoma risk: 60200, incision/excision thyroid cyst; 60260, Thyroidectomy, removal of all remaining thyroid tissue following previous removal of a portion of thyroid (completion or redo); 60254, Thyroidectomy, total or subtotal for malignancy with radical neck dissection; 60270, thyroidectomy, including substernal thyroid, sternal split or transthoracic approach; 60252, thyroidectomy, total or subtotal for malignancy, with limited neck dissection; 60271, thyroidectomy, including substernal thyroid, cervical approach. We excluded patients who underwent thyroidectomy for indications other than thyroid disease (e.g., laryngeal or hypopharyngeal cancer, parathyroid disease) by excluding the follow International Classification of Diseases (ICD)-10 codes: C16.0, C32.2, C32.9, C75.0, C77.0, D35.1, E21.0, E21.2, and E21.3.

### Data collection

The primary outcome variable was post-operative hematoma. Hematoma was defined as “postoperative bleeding at neck site or the development of a hematoma”. Note that “ecchymosis” or “bruising” alone at the surgical site did not qualify as hematoma. The secondary outcome variable was operative time. The exposure variable was use of alternative sources of cautery (Harmonic scalpel and LigaSure). In the NSQIP database, the use of a harmonic scalpel, LigaSure, or other vessel sealing device was indicated as “Yes—specifically noted” if it was specifically mentioned in the operative report. Based on previously reported literature and a-priori knowledge of risk factors for post-operative hematoma, and factors affecting operative time, we also extracted data on a variety of covariates. These included variables related to patient demographics, including age, sex, and race, as well as potentially confounding clinical variables, including presence of bleeding disorders, hypertension requiring medications, pre-operative platelet count, pre-operative international normalized ratio (INR), American Society of Anesthesiologists (ASA) class, and drain use.

### Statistical analysis

Patients with missing data or incomplete follow-up were excluded from the analysis. Descriptive statistics were used to report baseline patient characteristics, including proportions, means, and standard deviations (SDs). χ^2^ statistics were used to compare hematoma proportions between cautery groups. Univariable and multivariable logistic regression modelling were used to assess the predictors of post-operative hematoma. Effects estimates were reported as odds ratio (ORs) and 95% confidence intervals (CIs), comparing conventional and alternative sources of cautery. Univariable and multivariable linear regression models were used to assess predictors of operative time, comparing conventional and alternate sources of cautery. Multivariable models were constructed using explanatory variables chosen a priori based on clinical importance, and those that were observed to be highly significant in univariable models (p < 0.001). Otherwise, a p value of < 0.05 was used as the cut-off for statistical significance. All analyses were performed using SAS version 9.4 (SAS Institute, Cary, NC).

### Ethics approval and consent to participate

This study did not require institution ethical approval as NSQIP data exempt from The Ottawa Hospital REB as it is deidentified. A consent waiver is not relevant as all data is deidentified.

## Results

**Figure 1:**
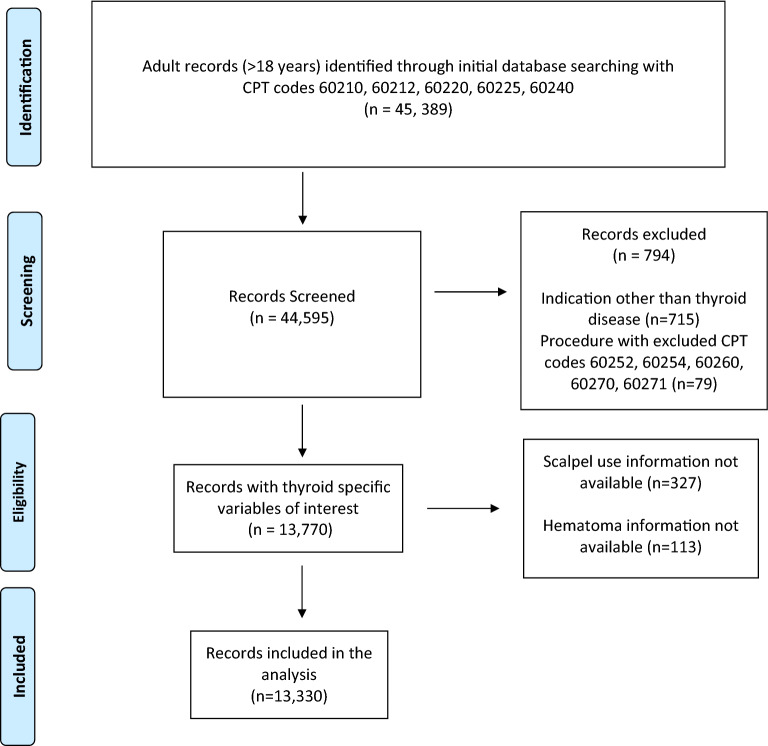
Record inclusion diagram. *n* number of patient records, *CPT* current procedural technology codes.

A total of 13,330 cases of total thyroidectomy, subtotal thyroidectomy, and completion thyroidectomy were identified from the ACS-NSQIP database. A total of 13,330 cases of total thyroidectomy, subtotal thyroidectomy, and completion thyroidectomy were identified from the ACS-NSQIP database. A total 45,389 adult patients had thyroidectomy listed as the principle procedure, based on previously defined CPT codes. Among these, 715 were excluded because the indication for the procedure was other than thyroid disease, and 79 were excluded for having undergone additional procedures which were deemed to be excludable a priori (CPT codes 60252, 60254, 60260, 60270, 60271). Among the remaining 44,595, only 13,770 had thyroid specific variables of interest. A further 327 were excluded because information regarding cautery type was unavailable, and 113 had missing data pertaining to the presence or absence of post-operative hematoma. The final population for analysis was comprised of 13,330 procedures. See Fig. 1.

Of the 13,300 procedures, 4342 (32.6%) were performed using conventional cautery, and 8988 (67.4%) were performed used alternative sources of cautery (Harmonic Scalpel or LigaSure). Descriptive statistics are summarized in Table 1.

**Table 1:** Demographics.

	All patients	Conventional cautery	Alternative cautery
N	13,330	4342 (32.57%)	8988 (67.43%)
**Gender**			
Female	10,568 (79.28%)	3338 (76.88%)	7231 (80.45%)
Male	2661 (19.96%)	1004 (23.12%)	1757 (19.55%)
**Age (years)**			
Age ≤ 70	11,954 (89.68%)	3849 (88.65%)	8105 (90.18%)
Age > 70	1376 (10.32%)	493 (11.35%)	883 (9.82%)

The overall reported rate of post-operative hematoma regardless of type of cautery used was 245/13,330 operations (1.8%). The overall rate of post-operative hematoma in operations using conventional cautery was 93/4342 (2.1%), and 152/8988 (1.7%) in those using alternative sources of cautery. The crude hematoma rate among operations using alternative sources of cautery was not significantly different than that in operations using conventional cautery (p = 0.07) (Table 2). The number needed to treat (NNT) to prevent one hematoma was 222.

**Table 2:** Rate of post-operative thyroid hematoma development in all patients and by type of cautery used.

Group	Rate of thyroid hematoma (%)
Overall (all patients)	1.8
Conventional cautery	2.2
Alternative sources of cautery	1.7

Univariable analyses demonstrated the following risk factors to be associated with significantly increased odds of post-operative hematoma: age, gender, body mass index (BMI), history of a bleeding disorder, history of using medication to treat hypertension, and ASA score (Table 3).

**Table 3:** Results of all variables by univariate analysis for post-operative hematoma.

Variable	ODDs ratio	95% confidence limits		p-value
Increase in age by 1 year^a^	1.0165	1.0077	1.0253	0.0002
> 70 vs. ≤ 70 in age	1.2644	0.864	1.8505	0.2273
Male vs. female^a^	1.6746	1.2707	2.2069	0.0003
Increase in BMI by 1	1.0138	0.9977	1.0301	0.0928
> 30 vs. ≤ 30 BMI^a^	1.2943	1.0044	1.6677	0.0461
Non-white vs. white	1.3148	0.9679	1.7861	0.0799
Yes vs. no bleeding disorder^a^	3.9064	2.0307	7.5148	< 0.0001
Yes vs. no meds for hypertension^a^	1.7475	1.3568	2.2506	< 0.0001
Increase in platelets by 1	0.9992	0.997	1.0013	0.4466
Increase in INR by 1	1.0463	0.4676	2.341	0.9124
≥ 3 vs. 1–2 ASA^a^	1.7017	1.3205	2.193	< 0.0001
Yes vs. no alternative scalpel use^a^	0.7859	0.6056	1.0199	0.0701
Yes vs. no drain use^a^	0.9601	0.7157	1.2881	0.786

^a^Indicates variables carried forward to the multivariable analysis.

Multivariable analysis demonstrated that, when compared to conventional hemostatic technique, alternate sources of cautery were associated with a statistically significant decrease in the odds of post-operative hematoma, OR 0.75 (95% CI 0.58–0.98) (p = 0.04). See Table 4 for the full results of the multivariable analysis for post-operative hematoma risk.

**Table 4:** Results of multivariate analysis for risk factors for post-operative hematoma.

Variable	ODDs ratio	95% confidence limits		p-value
Increase in age by 1 year	1.0066	0.9967	1.0167	0.1936
Male vs. female	1.5204	1.1485	2.0128	0.0034
> 30 vs. ≤ 30 BMI	1.1757	0.8992	1.5372	0.2367
Yes vs. no bleeding disorder	2.9982	1.5382	5.8439	0.0013
Yes vs. no meds for hypertension	1.3515	1.0043	1.8188	0.0468
≥ 3 vs. 1–2 ASA	1.3007	0.9763	1.7329	0.0724
Yes vs. no alternative scalpel use	0.7527	0.5779	0.9803	0.035
Yes vs. no drain use	0.8535	0.6334	1.15	0.2977

Multivariable analysis for operative time demonstrated that there was a statistically significant increase in operative time by 4.95 min (95% CI 2.45–7.45) (p = 0.0001) when alternative sources of cautery were used, compared to conventional cautery. See Table 5 for results of the univariable analysis for operative time, and Table 6 for results of the multivariable analysis for operative time.

**Table 5:** Results of univariate analysis for factors affecting operative time.

Variable	Estimate	95% confidence limits		p-value
Increase in age by 1 year^a^	− 0.1854	− 0.2537	− 0.117	< 0.0001
> 70 vs. ≤ 70 in age	− 4.594	− 7.9215	− 1.2665	0.0068
Male vs. female^a^	14.9983	12.5124	17.4842	< 0.0001
Increase in BMI by 1	0.7607	0.6265	0.895	< 0.0001
> 30 vs. ≤ 30 BMI^a^	9.1537	7.1191	11.1883	< 0.0001
Non-white vs. white^a^	− 17.71	− 20.177	− 15.243	< 0.0001
Yes vs. no bleeding disorder	5.0026	− 4.5661	14.5714	0.3055
Yes vs. no meds for hypertension^a^	6.3823	4.2986	8.466	< 0.0001
Increase in platelet by 1	0.0165	− 0.0015	0.0345	0.0727
Increase in INR by 1	− 0.5827	− 8.6304	7.465	0.8871
≥ 3 vs. 1–2 ASA^a^	12.1308	9.9993	14.2622	< 0.0001
Yes vs. no alternative scalpel use^a^	5.1727	3.0136	7.3318	< 0.0001
Yes vs. no drain use^a^	35.601	33.3493	37.8527	< 0.0001

^a^Indicates variables carried forward to the multivariate analysis.

**Table 6:** Results of multivariate analysis for factors affecting operative time.

Variable	Estimate	95% confidence limits		p-value
Increase in age by 1 year	− 0.3847	− 0.4666	− 0.3027	< 0.0001
Male vs. female	16.0787	13.4439	18.7135	< 0.0001
> 30 vs. ≤ 30 BMI	3.7975	1.5179	6.0771	0.0011
Non-white vs. white	− 12.4954	− 15.1293	− 9.8614	< 0.0001
Yes vs. no bleeding disorder	0.8624	− 9.3578	11.0826	0.8686
Yes vs. no meds for hypertension	2.558	− 0.0693	5.1854	0.0564
≥ 3 vs. 1–2 ASA	9.3123	6.7664	11.8582	< 0.0001
Yes vs. no alternative scalpel use	4.9485	2.4461	7.4508	0.0001
Yes vs. no drain use	31.388	28.8084	33.9677	< 0.0001

## Discussion

This retrospective risk-adjusted analysis using the National Surgical Quality Improvement Program (NSQIP) was completed to (1) compare post-operative hematoma rates between thyroid surgery using conventional cautery and alternative energy devices, and (2) compare operative times for the same. Within our data set, odds of post-operative hematoma were significantly lower in the thyroidectomy cases in which alternative sources of cautery were used. Operative time was longer by 4.95 min in the alternative energy device group, which was statistically significant, however may not be clinically significant.

Techniques for hemostasis in thyroid surgery have been compared and contrasted throughout the literature. The ideal technique is still controversial. While many operative and post-operative parameters have been examined at length including operative time, intra-operative blood loss, surgical completeness in oncologic surgery, cost-effectiveness, and some post-operative complications, post-operative hematoma rates have not been specifically studied as a primary outcome^6–8^. Post-operative hematoma rate is an important outcome to measure because it is a life-threatening complication post-thyroidectomy.

A systematic review and meta-analysis published in 2009 evaluated the hemostatic effects and safety of thyroidectomy performed using the LigaSure vessel-sealing device vs conventional vessel ligation^7^. Authors screened studies performed between 1996 and 2008; four randomized and five nonrandomized trials were included. No significant difference was found between the alternate energy device and conventional thyroidectomy for overall incidence of postoperative complications (OR 0.91; 95% CI 0.61–1.36), a composite variable which included hematoma, nerve damage, hypocalcemia, and incision infections. There was no statistical analysis done on hematoma rates alone. Raw numbers reported were two hematomas after thyroidectomy with conventional cautery vs three hematomas after thyroidectomy with LigaSure. Operative duration was significantly reduced in thyroidectomies performed using LigaSure, with a weighted mean difference of − 11.97 min (p < 0.001)^7^.

In 2010, there was a systematic review and meta-analysis published to evaluate hemostasis in thyroid surgery comparing the Harmonic Scalpel to other hemostatic techniques^6^. The authors screened 72 potential studies and included 12 prospective randomized control trials in the analysis. Again, hematoma rates were not presented as an individual outcome, but were reported as part of a composite variable of complications including: hypocalcemia, hypothyroidism, seroma and dysphonia. Raw hematoma rates were not reported. There was no significant difference in adverse events between Harmonic Scalpel and other hemostasis techniques (OR 0.77; 95% CI 0.53–1.11). Hemostasis was also measured by intraoperative blood loss and post-operative drain collections. Blood loss was significantly less with Harmonic Scalpel (decreased by 20.03 mL; 95% CI − 27.83 to − 12.22; p < 0.00001), but differences in drain outputs were not statistically significant. Operative time was reduced using the Harmonic Scalpel (mean operative time reduction was 22.67 min; 95% CI − 27.98 to 17.37; p < 0.00001)^6^.

Our current study findings are incongruent with previous literature reporting no significant difference in post-operative complication rates between these two techniques. Specifically, we found that hematoma rates were actually decreased when alternative sources of cautery were used. Our study is the first to analyse hematoma as a primary outcome, rather than including it in a composite variable with other post-operative complications. The ultimate clinical significance of this finding is debateable, though, given that rates of post-operative hematoma were low in both groups (2.1% in conventional hemostasis group, and 1.7% in alternative cautery group). The statistical significance is likely a result of our large population (13,330 total thyroid operations).

Our finding that the use of alternate sources of cautery increases operative time also differs from what has been reported to date. Based on our results, we cannot definitively conclude why our data does not reflect that of previous literature. However, several hypotheses exist, including: potential heterogeneity of the term ‘conventional’ cautery, a learning curve associated with the use of alternate sources which is not captured in our data, and the potential inherent risk of bias in previous studies that have been conducted using industry funding.

Our findings of differences in hematoma risk and operative time must not be considered in a vacuum. In particular, there is an ongoing movement towards cost-effectiveness in surgery. As a commonly performed procedure, thyroidectomy has been associated with substantial healthcare costs (hospital charges exceeded 2.5 billion US dollars in 2006)^12^. At our institution, the Harmonic Scalpel is $485.00 CAD per disposable handheld device, and the LigaSure is $315.00 CAD per disposable handheld device. Given our analysis found an overall increase in operative time using alternative sources of cautery, the discussion on cost effectiveness is relevant to explore given these devices are also more expensive than traditional methods of cautery. There have been two systematic reviews completed in recent years assessing the overall cost effectiveness of alternative sources of cautery in thyroidectomy. Cheng et al. looked at hospital costs associated with thyroidectomy performed with the Harmonic Scalpel compared to conventional cautery^13^. They concluded that compared to conventional techniques, the alternative energy device reduced total costs by 10% (p = 0.007), with a large portion of the overall savings derived from a reduction in operative costs (in part as a function of decreased operative time)^13^. The study was sponsored by Ethicon. A second systematic review and meta-analysis performed by Zhang et al. concluded there was no difference in total cost in-hospital (p = 0.08) between ultrasonic coagulator and conventional techniques^14^. Our study found that alternative sources of cautery were associated with statistically prolonged operative time, however, this increase of 4.95 min may not be clinically significant. Additionally, our study was not designed to determine cost-effectiveness and as such, that specific question would have to be addressed in a separate study. Our results do demonstrate that the use of alternate sources of cautery does not improve operative time.

### Limitations

Our data was derived from NSQIP, which is associated with certain inherent limitations. The key limitations of this study are: (1) potential confounding effects of variables not captured in the NSQIP database, (2) information bias from reliance on retrospective data, (3) the relatively recent addition of thyroid specific data to the database, limiting collection years to 2016–2018, (4) missing database information. With regards to potentially unmeasured confounding, NSQIP does not collect data on anticoagulant use, limiting our ability to control for this variable. We did, however, control for patient platelet counts, international normalized ratios (INRs), and bleeding disorders. By controlling for these variables, we were likely able to capture most patients at increased risk of bleeding, with the exception of those on direct oral anticoagulants (DOACs). Though we do not have reason to believe that there would be significant proportions of patients in either exposure group at increased risk of bleeding due to DOAC use, we acknowledge our inability to explicitly control for anticoagulation as a limitation. In addition, though NSQIP does collect data on other potentially relevant variables such as alcohol use and trainee level of supervision, the level of completeness of these variables prevented us from controlling for these variables in our analysis. In total, 30,825 patients in our study sample lacked information pertaining to thyroidectomy-specific variables, and a further 440 patients out of the 13,770 that had data pertaining to thyroid specific variables were excluded from the analysis due to pertinent missing data (327 patients lacking information regarding cautery type, and 113 lacking data on whether or not hematoma occurred). This limitation was unfortunately unavoidable within the constraints of the completeness of data contained within the NSQIP database. Lastly, our analysis showed an increase in operative time using alternative sources of cautery, however there are many unmeasured factors that may have affected this result including operative experience and case difficulty, so the direct clinical relevance of this finding is unclear.

### Strengths

Despite the above-mentioned limitations, this study derives strength from the quality of the NSQIP data source, which provided a robust sample size, and reliable data given rigorous data collection and auditing processes. With such a large sample of thyroidectomy procedures to analyze, we were able to detect statistically significant differences in hematoma rates and operative times between with a degree of precision. NSQIP has contributions from centers world-wide, strengthening the generalizability of our results.

## Conclusion

In summary, this retrospective risk-adjusted analysis using the National Surgical Quality Improvement Program (NSQIP) found a statistically significant decrease in post-operative hematoma risk in thyroid surgery performed using alternative sources of cautery compared to traditional sources. Additionally, we found that the use of these devices was associated with a statistically significant increase in operative time of 4.95 min. The clinical significance of the latter finding must be interpreted with caution. The implications of these findings on the cost-effectiveness of these devices is an area of research to be further explored.

## Data Availability

This study used NSQIP data.

## Abbreviations

ACS-NSQIP: American College of Surgeons National Surgical Quality Improvement Program
INR: International normalized ratio
ASA: American Society of Anesthesiologists
CPT: Current procedural technology
ICD: International Classification of Diseases
SD: Standard deviation
ORs: Odds ratio
CIs: Confidence intervals
BMI: Body mass index
DOACs: Direct oral anticoagulants
